# Electrotherapy as treatment for chemotherapy-induced peripheral neuropathy — a randomized controlled trial

**DOI:** 10.3389/fneur.2024.1451456

**Published:** 2024-12-24

**Authors:** Robert Sassmann, Simon Peter Gampenrieder, Florian Rieder, Tim Johansson, Gabriel Rinnerthaler, Vanessa Castagnaviz, Kathrin Lampl, Jürgen Herfert, Yvonne Theres Kienberger, Maria Flamm, Dagmar Schaffler-Schaden, Richard Greil

**Affiliations:** ^1^Institute of Physical Medicine and Rehabilitation, Paracelsus Medical University Salzburg, Salzburg, Austria; ^2^IIIrd Medical Department with Haematology, Medical Oncology, Haemostaseology, Infectiology and Rheumatology, Oncologic Center, Paracelsus Medical University Salzburg, Salzburg, Austria; ^3^Salzburg Cancer Research Institute (SCRI) - Laboratory for Immunological and Molecular Cancer Research (LIMCR) and Center for Clinical Cancer and Immunology Trials (CCCIT), Salzburg, Austria; ^4^Cancer Cluster Salzburg, Salzburg, Austria; ^5^Institute of General Practice, Family Medicine and Preventive Medicine, Paracelsus Medical University, Salzburg, Austria; ^6^Salzburg Regional Health Fund (SAGES), Salzburg, Austria; ^7^Division of Clinical Oncology, Department of Internal Medicine, Medical University of Graz, Graz, Austria; ^8^Red Bull Athlete Performance Center, Thalgau, Austria

**Keywords:** HTEMS, TENS, high tone therapy, QLQ-CIPN20, QLQ-C30

## Abstract

**Background:**

Electrotherapy has been investigated in chronic pain and diabetic peripheral neuropathy, however prospective trials in patients with chemotherapy-induced peripheral neuropathy (CIPN) are scarce.

**Methods:**

Fifty-one patients with CIPN ≥ grade 1 subsequent to receiving platinum- and/or taxane-based chemotherapy types were randomized to 8 weeks of high tone external muscle stimulation (HTEMS) or transcutaneous electrical nerve stimulation (TENS). The primary outcome were changes in the EORTC-QLQ-CIPN20 questionnaire. Secondary outcomes included clinical examinations, a classification of CIPN according to CTCAE v 4 and the EORTC-QLQ-C30 questionnaire. A control group (n = 17) receiving no intervention was recruited retrospectively.

**Results:**

The EORTC-QLQ-CIPN20 sensory and motor scales improved in both intervention groups (TENS: −12.3pts and − 8.2pts; HTEMS: −14.7pts and − 8.2pts) with no significant changes in the control group −3.3pts; −2.8pts. The changes in the sensory scale differed significantly between the HTEMS and the control group. In the EORTC-QLQ-C30 questionnaire, there was a significant improvement for physical functioning in the HTEMS group only (+7.9pts) with no between group differences. CIPN classification according to CTCAE v4 improved significantly in both intervention groups.

**Conclusion:**

Home-based electrotherapy with HTEMS or TENS were successful in improving CIPN-related sensory impairment and could therefore provide a powerful treatment for this side-effect of chemotherapy.

**Clinical trial registration:**

https://clinicaltrials.gov/ct2/show/NCT03978585

## 1 Introduction

Continuous advancements in diagnosis and treatment of cancer lead to rising numbers of cancer survivors and to increasing life expectancy (1). However, the progress in systemic therapy also brings challenges in the management of side effects. Chemotherapy-induced peripheral neuropathy (CIPN) is one major complication of several anti-cancer therapies, especially of taxanes and platinum salts (2). Additionally, the problems may increase with novel drug types like antibody-drug conjugates and checkpoint inhibitors (3, 4). In a meta-analysis of studies investigating taxanes and platinum salts, the average prevalence of CIPN was >60% in the first 3 months after cessation of chemotherapy and about 30% after 6 months and beyond (5). The severity of CIPN depends on the chemotherapeutic agent, the cumulative dose, and the treatment regime. Symptoms usually begin during chemotherapy at a lower intensity and tend to increase if chemotherapy is continued. As a result, dose reductions and ultimately discontinuation of chemotherapy are frequently necessary in order to avoid high-grade CIPN, that hampers daily activities and diminishes quality of life (6). Unfortunately, there is still no effective pharmacological or non-pharmacological treatment of CIPN available. Several approaches with antidepressants, antiepileptics, magnesium, calcium, vitamins E and B6, glutamine, glutatione, N-acetyl-ysteine, omega-3 fatty acids, alpha lipoic acid, topical ketamine, acupuncture, or magnetic field therapy failed to show reproducible and significant relief of symptoms (7–9).

Electrotherapy is a potential approach for alleviating symptoms in this population. Transcutaneous electrical nerve stimulation (TENS) has shown analgesic effects in various chronic pain conditions by increasing opioid receptor activation and restoring central inhibition (10–13). A single-arm, uncontrolled trial including 29 patients with CIPN showed that 6 weeks of TENS reduced sensory and motor impairments as well as pain (14). However, high level evidence is missing (15). Similarly, treatment with high-tone external muscle stimulation (HTEMS) seems a promising approach in the therapy of CIPN. Compared to TENS, HTEMS works with higher frequencies and might therefore also enhance blood circulation and cellular metabolism rather than only suppressing pain perception (16). This method is successfully used in the treatment of diabetic neuropathy (17, 18) and shows better results in the reduction of pain compared to TENS (16). To the best of our knowledge, there is one very recent (2024) placebo-controlled trial using HTEMS as treatment for CIPN indicating its potential by improving paresthesia and mental stress after 3 weeks of electrotherapy with no changes for the placebo group. However, this study was underpowered (*n* = 7 per group) and changes in sensory or motor impairments assessed with the EORTC QLQ-CIPN20 questionnaire did not reach significance (19). Hence, the objective of this randomized controlled clinical trial was to investigate the effectiveness of home-based electrical therapy in the treatment of CIPN. We hypothesized that both interventions (HTEMS and TENS) would mitigate CIPN symptoms and increase quality of life, with superior results for HTEMS.

## 2 Materials and methods

### 2.1 Setting

This was a single-blinded, randomized controlled trial with an observation time of 8 weeks. The trial was conducted at the University Hospital Salzburg from September 2019 until March 2023. The study protocol was approved by the ethics committee of Salzburg County (ID 415-E/2376/7–2018). All processes were performed in accordance with the 1964 Helsinki declaration and all patients gave their written informed consent. The study was registered in Clinical Trials, available at https://clinicaltrials.gov/ct2/show/NCT03978585. The study protocol was previously published in detail (20). However, after publication of the protocol the following amendments have been made: (1) in addition to patients with breast and colorectal cancer, individuals with other types of cancer were allowed to participate; (2) patients with CIPN ≥ grade 1 at baseline were included and (3) a control group fulfilling the same inclusion and exclusion criteria, but receiving no intervention for CIPN was recruited retrospectively. The reporting of this clinical trial follows the CONSORT guidelines (see CONSORT checklist in Supplementary material S5).

### 2.2 Study-flow

For the original study, CIPN patients were recruited, randomized and treated either with HTEMS or TENS therapy from September 2019 until October 2021. The control group, where patients received no intervention, was recruited in retrospect from July 2022 to March 2023.

### 2.3 Patient identification and recruitment

All patients receiving systemic tumor treatment at the IIIrd Medical Department of the Paracelsus Medical University Salzburg underwent screening for neuropathy complaints by using a standardized admission form or by collecting medical history orally. For inclusion, patients had to have completed chemotherapy with a taxane or platinum salt for a confirmed invasive cancer 4 to 24 weeks before, have a clinical diagnosis of CIPN ≥ grade 1 according to Common Terminology Criteria for Adverse Events version 4 (CTCAE v 4), be at least 18 years of age and have an Eastern Cooperative Oncology Group (ECOG) performance score of 0 to1. The CTCAE v 4 questionnaire includes the limitations of the activities of daily living and has 5 categories: 0 = no impairment, 1 = loss of deep tendon reflexes and paresthesia, 2 = limiting instrumental activities of daily living, 3 = limiting self-care ADL, 4 = life-threatening consequences. Exclusion criteria contained an ongoing or planned treatment with antitumor treatments with potentially neurotoxic side effects, preexisting peripheral neuropathy, peripheral arterial occlusive disease > grade 1, skin conditions preventing proper application of electrodes or implanted medical electronic devices (e.g., pacemaker).

### 2.4 Randomization and blinding

Participants were randomly allocated to either HTEMS or TENS. Randomization was performed centrally. The random allocation sequence was generated using the random number generator available online.^1^ Subjects were stratified according to the respective chemotherapeutic agent: taxane or platin. Physicians responsible for the clinical examinations and outcome assessment were blinded. Due to the technical design of the intervention, participants and device instructors could not be blinded. Patients in the control group were not randomized or blinded and did not receive any electrotherapy for CIPN symptoms. For this group, the statistician analyzing the data was blinded to the patient’s allocation.

### 2.5 Intervention and control

Participants of the intervention groups received instructions on the proper use of the electrical device and the first treatment under supervision. The further applications were carried out at home. After 1 week of use, a therapist called the patients to ensure proper use. Patients of both groups were instructed to use the electrical device daily for at least 30 min for 8 weeks. The minimum requirement of use for the per protocol analysis was at least 5 days a week corresponding to a total usage time of ≥1,200 min. To ensure the minimum requirement, frequency and duration of use were recorded in a diary filled out by the patients and on the electrical device.

HTEMS was administered using a HiTOP 191 device (gbo Medizintechnik, Rimbach, Germany). The conductive rubber electrodes were placed on the lower limbs (one at the calf and one on the sole of the foot). If the hands were also affected, patients were additionally allowed to perform the electrical therapy on the hands with electrodes placed on the frontal side of the forearm and on the back of the hand. The principle of HTEMS is based on muscle contraction in intervals. An interval comprised 3 sec of ramp-up time (where intensity increases to the pre-set maximum level), followed by 3 sec of holding time (where intensity remains at maximum), and finally, 3 sec of pause (with no stimulation). The applied frequencies varied in the same predefined order from 4,096 to 32,768 Hertz over three octaves in 72 quarter-tone steps of 1 sec each for each patient. The maximum intensity of the stimulation was initially set by a medical technician to a level that elicited tolerable muscle contractions without causing any pain or discomfort and was continually adjusted by the patient in order to maintain this effect.

Patients in the TENS group placed the rubber electrodes of the electric device (DoloBravo, MTR GmbH, Berlin, Germany) on the same body areas as described for the HTEMS electrodes. The manufacturer’s predefined applied frequency was 80 Hertz. The maximum intensity of the stimulation was set the same way as described for the HTEMS therapy.

A control group was recruited retrospectively to control for time-dependent symptom relief and to avoid overestimation of intervention effects. They completed the EORTC-QLQ-CIPN20 and EORTC-QLQ-C30 questionnaires twice, with an interval of 8 weeks and did not receive any electrotherapy within this period.

### 2.6 Outcomes

All outcome parameters were evaluated at baseline (T0) and at the end of the study, after 8 weeks of treatment (T1). The primary endpoint was the improvement in the disease specific EORTC-QLQ-CIPN20 questionnaire. This questionnaire contains 20 items assessing sensory (9 items), motor (8 items), and autonomic symptoms (3 items), using a 4-point Likert scale (1 = “not at all,” 2 = “a little,” 3 = “quite a bit,” and 4 = “very much”). All scale scores are linearly converted to a 0 to 100 scale (0 = no sensory impairment, 100 = worst sensory impairment) (21). A difference of ≥5.9 points was considered clinically significant (22). Secondary endpoints were improvements of the patient quality of life (EORTC-QLQ-C30) and the classification of CIPN grade according to National Cancer Institute Common Terminology Criteria for Adverse Events (CTCAE) version 4 (23). Clinician-reported secondary outcomes were assessed in the HTEMS and the TENS group with a standardized clinical test battery containing the following assessments: Vibration sensibility measured with a semi-quantitative tuning fork (24), Achilles and patellar tendon reflexes (25), temperature sensibility (26), perception of touch, by symmetrically stroking the patient’s thighs, lower legs and feet with the physician’s fingers (27) and strength of the lower leg muscles (by performing toe standing/walking and heel standing/walking on both feet; possible, not possible). A detailed description of the clinical assessments can be found in the Supplementary Tables S4–S6.

Patients in the control group did only complete the EORTC-QLQ-CIPN20 and EORTC-QLQ-C30 questionnaires. No other outcome parameters were recorded.

### 2.7 Statistical analysis

Sample size was calculated *a priori* for a power of 80%, *α* = 0.05 and *β* = 0.20, proposing a 5.9 point difference in pre-post changes of the EORTC-CIPN20 scores between both treatment groups. Using an estimated standard deviation of 5.5, a sample size of 42 patients (21 per arm) would be required. Considering an estimated drop-out rate of 15%, we defined a recruitment goal of 50 patients.

The analysis for the primary endpoint was based on the intention-to-treat principle, secondary endpoints were analyzed per protocol. Normal distribution of data was assessed by the Shapiro–Wilk test. Between-group differences at baseline were analyzed using a student’s t-test for independent and normal distributed data and a Mann–Whitney U-Test or a Chi-square test for nonparametric data. Within-group differences were calculated using a student’s t-test for paired samples if data was normally distributed, otherwise a Wilcoxon signed-rank test was used. *p*-values were Bonferroni corrected. Pre-post changes between groups were analyzed using a Kurskal Wallis test for the EORTC-QLQ-CIPN20 and EORTC-QLQ-C30 questionnaires (all data not normally distributed). If a significant effect was found, Mann–Whitney U-Tests were performed post-hoc with Bonferroni corrected *p*-values. Within-group differences for all other secondary endpoints were analyzed using a Wilcoxon signed-rank test or a McNemar test, respectively. Pre-post differences between groups for the CTCAE v4 CIPN grade were analyzed using a Mann–Whitney U-Test test. All tests were 2-tailed, and a 5% probability level was considered as significant. All statistical analyses were performed with IBM SPSS Statistics 23.0 (IBM, Enhingen, Germany). Figures were created using GraphPad prism v.9 (GraphPad Software, Boston, United States).

## 3 Results

**Figure 1:**
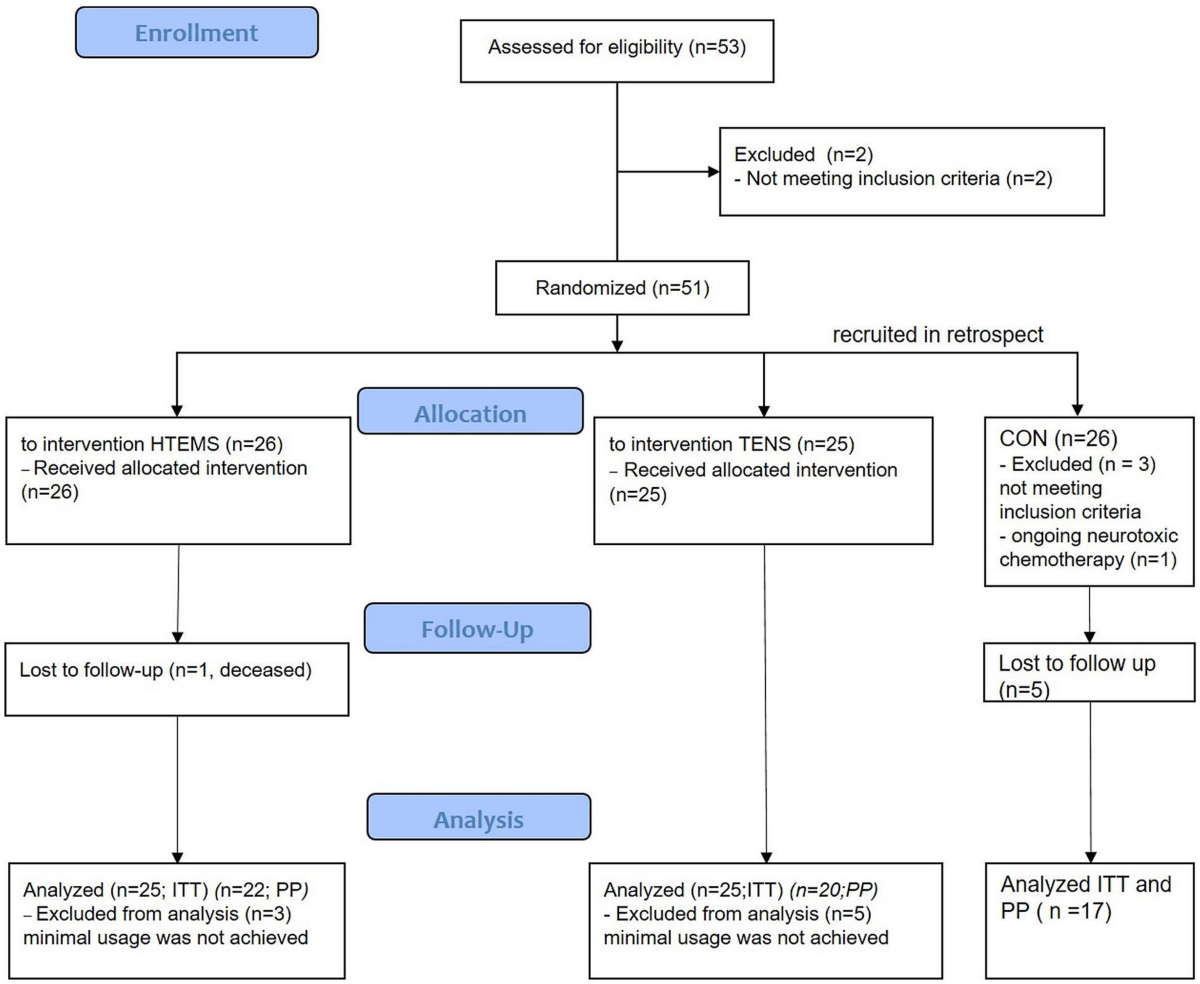
Flow diagram of the study. HTEMS: high tone external muscle stimulation; TENS: transcutaneous electrical nerve stimulation, both groups recruited and treated from September 2019 until October 2021; CON: control group, recruited in retrospect from July 2022 to March 2023; ITT: intention to treat analysis for primary outcome; PP: per protocol analysis for secondary outcomes.

In total, 51 patients were included between September 2019 and October 2021 and randomized to the TENS or HTEMS group. One patient in the HTEMS group died during the study period due to his cancer and was not included in the intention-to-treat analyses for the primary outcome EORTC-QLQ-CIPN20 questionnaire (*n* = 50). Three patients in the HTEMS group and five patients in the TENS group were not included in the per-protocol analysis for secondary outcomes (*n* = 42), because they did not achieve the minimal total usage time for electrical therapy (≥ 1,200 min). The retrospectively recruited control group (CON) consisted of 10 male and 7 female (in total *n* = 17) patients. The study flow and the baseline characteristics of patients are demonstrated in Figure 1 and Table 1.

**Table 1:** Baseline tumor and patient characteristics.

	HTEMS *n* = 25	TENS *n* = 25	Controls *n* = 17	*p*- value
Median age [years] (range)	63 (36–89)	69 (24–81)	63 (47–79)	0.748
Age < 60y	6 (24%)	11 (44%)	4 (23%)	
Gender				0.832
Male	10 (40%)	12 (48%)	10 (59%)	
Female	15 (60%)	13 (52%)	7 (41%)	
Tumor entity				0.922
Breast cancer	11 (44%)	10 (40%)	5 (29%)	
Colorectal cancer	4 (16%)	6 (24%)	5 (29%)	
Esophageal cancer	2 (8%)	3 (12%)	2 (12%)	
Pancreatic cancer	1 (4%)	4 (16%)	2 (12%)	
Gastric cancer	2 (8%)	2 (8%)	2 (12%)	
Other	5 (20%)	0 (0%)	1 (6%)	
Tumor AJCC stage				
I	3 (12%)	3 (12%)	2 (12%)	0.543
II	6 (24%)	8 (32%)	4 (23%)	
III	8 (32%)	2 (8%)	5 (29%)	
IV	8 (32%)	12 (48%)	6 (35%)	
Grade				0.910
1–2	12 (48%)	11 (44%)	6 (35%)	
3	7 (28%)	10 (40%)	6 (35%)	
Unknown	6 (24%)	4 (16%)	5 (30)	
Therapeutic setting				
Curative	16 (64%)	15 (60%)	11 (65%)	0.939
Palliative	9 (36%)	10 (40%)	6 (35%)	
Neurotoxic chemotherapy*				0.049
Taxane	12 (48%)	11 (44%)	6 (35%)	
Platinum^#^	8 (32%)	9 (36%)	8 (47%)^#^	
Taxane and Platinum	5 (20%)	5 (20%)	3 (18%)	
Mean duration of neurotoxic chemotherapy [days] (SD)	104 (34)	134 (101)	143 (72)	0.193
Median duration [days] (range)	105 (140)	89 (477)	170 (270)	
Early discontinuation because of CIPN	10 (40%)	8 (32%)	3 (18%)	0.308
Mean time after ending of neurotoxic chemotherapy [days] (SD)	87(32)	98 (39)	97 (31)	0.473
Dose reduction of neurotoxic chemotherapy				0.193
Yes	8 (32%)	10 (40%)	6 (35%)	
No	17 (68%)	15 (60%)	11 (65%)	
BMI at baseline				
Median (range)	24.2 (19–36)	25.8 (19–35)	23.7 (17–31)	0.215
< 20	1 (4%)	2 (8%)	2 (12%)	
20–25	14 (56%)	15 (60%)	10 (59%)	
> 25	10 (40%)	8 (32%)	5 (29%)	
Variation of BMI during neurotoxic chemotherapy [mean] (SD)	−1.9 (7.1)	−2.0 (6.7)	−2,8 (7.3)	0.230
Increase	10 (40%)	11 (44%)	5 (30%)	
Loss ≤10%	12 (48%)	10 (40%)	8 (47%)	
Loss of ≥10%	3 (12%)	4 (16%)	4 (23%)	
Known Diabetes/Pre-Diabetes (y/n)				0.180
Diabetes	6 (24%)	4 (16%)	7 (41%)	
No diabetes	19 (76%)	21 (84%)	10 (59%)	
Therapy during electrotherapy				
Yes	15 (60%)	14 (56%)	13 (77%)	
No	10 (40%)	11 (44%)	4 (23%)	
Chemotherapy†	6 (24%)	2 (8%)	7 (41%)	
Chemotherapy† and targeted therapy or immunotherapy	4 (16%)	4 (16%)	3 (18%)	
Endocrine therapy	2 (8%)	2 (8%)	1 (6%)	
Targeted therapy ± endocrine therapy	3 (12%)	6 (24%)	2 (12%)	

AJCC, American Joint Committee on Cancer; BMI, body mass index; HTEMS, high tone external muscle stimulation; TENS, transcutaneous electrical nerve stimulation; SD, standard deviation; * stratification factor for both intervention groups; # significant baseline differences between the control and the intervention groups; †Non-neurotoxic chemotherapies only were allowed (e.g. capecitabine, 5-fluorouracil, and irinotecan etc).

**Figure 2:**
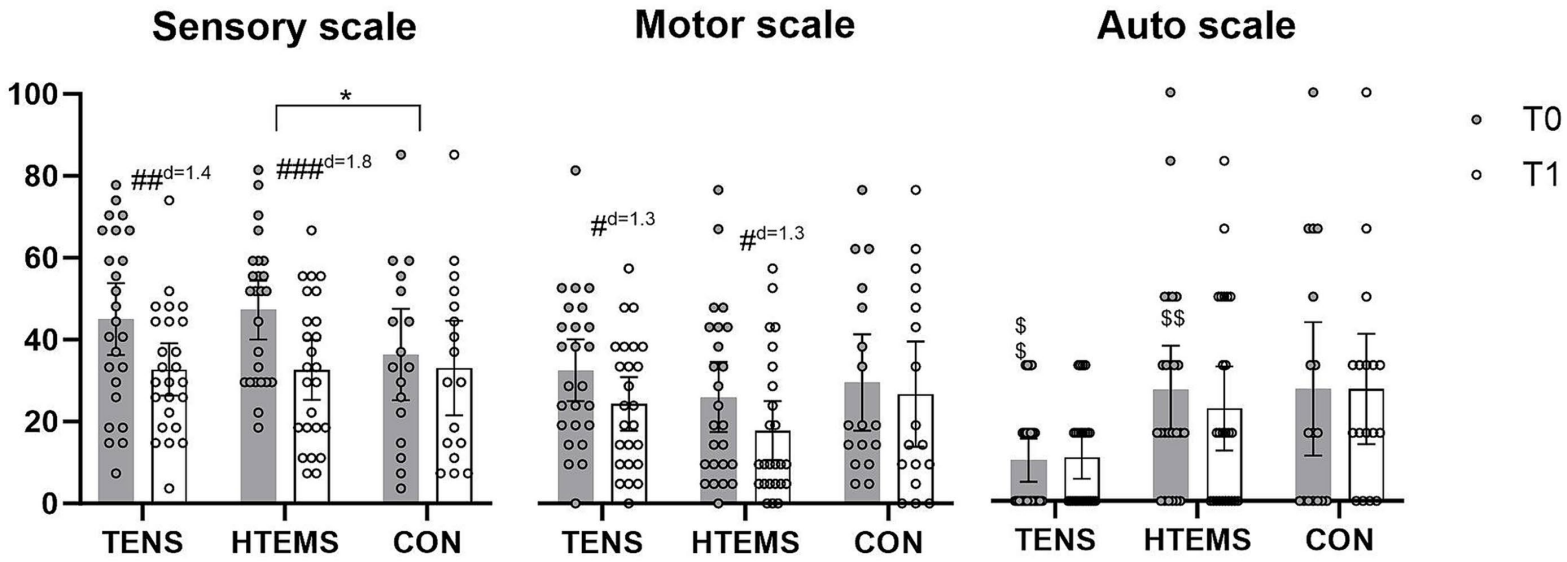
EORTC-QLQ-CIPN20 questionnaires before (T0, gray) and after (T1, white) the intervention in the transcutaneous electrical nerve stimulation (TENS), the high-tone external muscle stimulation (HTEMS) and the control (CON) groups, respectively. Significant within group differences: # *p* < 0.05, ## *p* < 0.01, ### *p* < 0.001; significant between group differences: * *p* < 0.05; significant baseline differences: $$ *p* < 0.01; d: effect size Cohan’s d. Values are presented as means and 95% confidence intervals with plotted individual values.

There were no baseline differences between groups except for the distribution of type of neurotoxic chemotherapy (*p* = 0.049) with higher percentage of platinum in the control group (Table 1). Baseline EORTC-QLQ-CIPN20 values in the autonomic scale also differed significantly between the three groups: patients in the TENS group had lower baseline values than patients in the two other groups. Baseline values for sensory scale showed a large numerical difference between the control and both intervention groups (CON 36 vs. TENS 47 vs. HTEMS 45), however this was not statistically significant (*p* = 0.213) (Table 2; Figure 2).

**Table 2:** EORTC QLQ CIPN20.

	HTEMS *n* = 25			TENS *n* = 25			Controls (CON) *n* = 17			
	T0	T1	Δ (CI)	T0	T1	Δ (CI)	T0	T1	Δ (CI)	Between group differences^1^
Sensory scale	45.0 ± 21.2	32.7 ± 15.4	−12.3** ^d = 1.4^ (−19.6; −5.0)	47.3 ± 17.5	32.6 ± 17.7	−14.7*** ^d = 1.8^ (−21.5; −7.8)	36.4 ± 21.7	33.2 ± 22.4	−3.3 (−9.7; 3.1)	TENS vs. CON *p* = 0.0204;
										HTEMS vs. CON *p* = 0.039
										TENS vs. HTEMS *p* = 1.0
Motor scale	32.4 ± 18.2	24.2 ± 15.7	−8.2* ^d = 1.3^ (−13.5; −2.6)	25.9 ± 20.6	17.7 ± 17.3	−8.2* ^d = 1.3^ (−13.7; −2.7)	29.4 ± 22.7	26.6 ± 24.7	−2.8 (−7.9; 2.3)	TENS vs. CON *p* = 1.0
										HTEMS vs. CON *p* = 0.726;
										TENS vs. HTEMS *p* = 1.0
Autonomic scale	10.0 ± 12.7^$$^	10.7 ± 12.6	0.7 (−4.8; 6.1)	27.3 ± 25.9^$$^	22.7 ± 24.9	−4.7 (−14.7; 5.4)	27.5 ± 31.7	27.5 ± 26.3	0.0 (−9.6; 9.6)	

Significant different baseline values compared to HTEMS $$ *p* < 0.01; significant within group differences ***p < 0.001; **p < 0.01; *p < 0.05; 1 Mann–Whitney-U-Test, d Cohan’s d for effect size.

### 3.1 Feasibility

Eight patients (16%; 3 in the HTEMS group and 5 in the TENS group) reported adverse events related to the therapy. Two patients in the HTEMS group complained about pain caused by too high intensity and one reported an increase of symptom intensity. Three patients of the TENS group reported that the electrodes were overly adhesive, one patient complained about an increase of symptom intensity and for one patient the electrodes could not be ideally fixed on the skin. There was no difference in therapy compliance between TENS and HTEMS groups. Eighty-eight percent of patients randomized to HTEMS and 80% of patients randomized to TENS patients fulfilled the minimal duration of electrical therapy (≥ 1,200 min).

### 3.2 Primary endpoint

The primary endpoint for this study were changes in the EORTC-QLQ-CIPN20 questionnaire. Values of patients in the TENS and HTEMS groups improved significantly in the sensory (TENS: −12.3 ± 17.7, *p* = 0.006; HTEMS: −14.7 ± 16.5, *p* < 0.001) and motor scale (TENS: −8.2 ± 12.9, *p* = 0.012; HTEMS; −8.2 ± 13.4, *p* = 0.015), but not for the autonomic scale (Figure 2; Supplementary Table S2). There were no significant changes in the control group for any scale (sensory scale: −3.3 ± 12.4, *p* = 0.294, motor scale: −2.8 ± 10.0, *p* = 0.264 autonomic scale: 0.0 ± 18.6, *p* = 1.000). According to the Kurskal-Wallis test the groups differed significantly when comparing pre-post differences in the sensory scale (*p* = 0.048). By performing pairwise comparisons post-hoc, there were significant differences between the HTEMS and control group (*p* = 0.039). There were no further between group differences (Figure 2; Table 2).

### 3.3 Secondary endpoints

All secondary endpoints were analyzed after the per protocol principle. Twenty, 22 and 17 patients completed the EORTC-QLQ-C30 questionnaire in the TENS, the HTEMS and the control groups, respectively. Results of the EORTC-QLQ-C30 questionnaire are presented in Tables 3, 4. There was a tendency for an increase in the global health status (+8.7 ± 15.7, *p* = 0.054) and a significant improvement for physical functioning (7.9 ± 11.8, *p* = 0.018) from T0 to T1 in the HTEMS group only (Table 3). There were no further within group differences. Pre-post differences did not differ between groups in any variable (Tables 3, 4).

**Table 3:** EORTC QLQ C30 symptom scales.

	TENS (*n* = 20)			HTEMS (*n* = 22)			Controls (*n* = 17)		
Symptom scales	T0	T1	Δ (CI)	T0	T1	Δ (CI)	T0	T1	Δ (CI)
Fatigue	38.9 ± 29.8	37.2 ± 31.7	−1.7 (−13.7;10.4)	43.3 ± 37.1	35.4 ± 29.6	−8.1 (−18.3;2.1)	52.3 ± 26.3	46.4 ± 28.1	−5.9 (−18.8;7.1)
Pain	38.3 ± 33.4	20.8 ± 31.0	−17.5 (−32.8;-2.2)	30.3 ± 36.6	24.2 ± 29.0	−6.1 (−15.6;3.5)	26.5 ± 20.5	25.5 ± 28.9	−1.0 (−17.2;15.2)
Nausea and vomiting	3.3 ± 6.8	5.0 ± 10.9	1.7 (−2.6;6.0)	9.1 ± 24.0	7.5 ± 18.3	−1.5 (−5.4;2.4)	12.7 ± 25.4	8.8 ± 18.7	−3.9 (−15.9;8.0)
Dyspnoea	10.0 ± 15.7	10.0 ± 21.9	0.0 (−11.3;11.3)	13.6 ± 28.5	15.2 ± 26.7	1.5 (−7.0;10.0)	27.5 ± 29.4	23.5 ± 25.7	−3.9 (−15.9;8.0)
Insomnia	30.0 ± 35.7	33.3 ± 37.5	3.3 (−9.0:15.6)	39.4 ± 40.7	27.3 ± 31.9	−12.1 (−31.8;7.5)	41.2 ± 34.4	35.3 ± 27.6	−5.9 (−22.2;10.4)
Appetite loss	16.7 ± 27.6	16.7 ± 29.6	0.0 (−7.2;7.2)	27.3 ± 39.4	22.7 ± 33.2	−4.5 (−27.7;4.2)	35.3 ± 41.6	23.5 ± 32.8	−11.8 (−27.4;4.2)
Constipation	20.0 ± 31.3	11.7 ± 24.8	−8.3 (−18.3;1.6)	9.1 ± 25.6	6.1 ± 19.6	−3.0 (−7.4;1.3)	19.6 ± 29	13.7 ± 23.7	−5.9 (−16.8;5.0)
Diarrhea	15.0 ± 29.6	15.0 ± 27.5	0.0 (−14.3;14.3)	19.7 ± 32.0	7.6 ± 22.8	−12.1 (−25.5;1.2)	39.2 ± 31.7	23.5 ± 36.8	−15.7 (−31.9;0.5)
Financial difficulties	11.7 ± 24.8	5.0 ± 16.3	−6.7 (−17.5;4.2)	15.2 ± 28.6	10.6 ± 26.0	−4.5 (−9.7;0.64)	17.6 ± 23.9	17.6 ± 23.9	0.0 (−8.6;8.6)

HTEMS, high tone external muscle stimulation; TENS, transcutaneous electrical nerve stimulation.

**Table 4:** EORTC QLQ C30 global health status and functional scales.

	TENS (*n* = 20)			HTEMS (*n* = 22)			Controls (*n* = 17)			
	T0	T1	Δ (CI)	T0	T1	Δ (CI)	T0	T1	Δ (CI)	Between group differences^1^
Global health status	55.8 ± 24.0	62.9 ± 20.9	7.1 (−3,1;17.2)	52.3 ± 13.7	61.0 ± 17.7	8.7 (1.7;15.7)	61.3 ± 17.4	60.8 ± 22.6	−0.5 (−12.6;11.6)	
Physical functioning	72.7 ± 20.5	77.0 ± 22.8	4.3 (−2.4;11.1)	72.7 ± 24.8	80.6 ± 20.9	7.9^2^*^r = 0.6^ (2.7;13.1)	68.2 ± 22.9	67.8 ± 20.2	−0.4 (10.3;9.6)	HTEMS vs. CON *p* = 0.432
										HTEMS vs. TENS *p* = 1.000
Role functioning	59.2 ± 23.7	64.2 ± 29.3	5.0 (−8.9;18.9)	59.1 ± 37.7	68.2 ± 30.4	9.1 (−7.0;25.2)	50.0 ± 26.4	58.8 ± 32.3	8.8 (−6.4;24.0)	
Emotional functioning	78.8 ± 21.5	80.8 ± 20.1	2.1 (−7.1;11.3)	67.0 ± 26.7	75.0 ± 20.1	8.0 (−1.3;17.2)	60.3 ± 25.3	65.2 ± 29.8	4.9 (−6.8;16.6)	
Cognitive functioning	80.0 ± 27.4	80.1 ± 21.1	0.8 (−8.5;10.1)	81.8 ± 27.2	81.1 ± 27.4	−0.8 (−8.1;6.6)	65.7 ± 34.6	70.6 ± 22.5	4.9 (−5.9;15.7)	
Social functioning	69.2 ± 29.3	77.5 ± 22.5	8.3 (−3.9;20.6)	63.6 ± 34.4	70.5 ± 27.7	6.8 (−5.2;18.8)	63.7 ± 27.8	70.6 ± 24.0	6.9 (−7.4;21.1)	

Significant within group difference *p < 0.05; ^1^Mann–Whitney-U-Test; ^2^Wilkoxon-Test; ^r^Pearson’s r as effect size.

There were significant improvements in the CIPN grading according to CTCAE v4 from T0 to T1 in both intervention groups (TENS: from 3 to 1, *p* = 0.004; HTEMS from 2 to 1, *p* = 0.012) with no between group differences (Supplementary Figure S3). There were no within or between group differences in any clinical assessment (Supplementary Tables S2–S4).

## 4 Discussion

**Figure 3:**
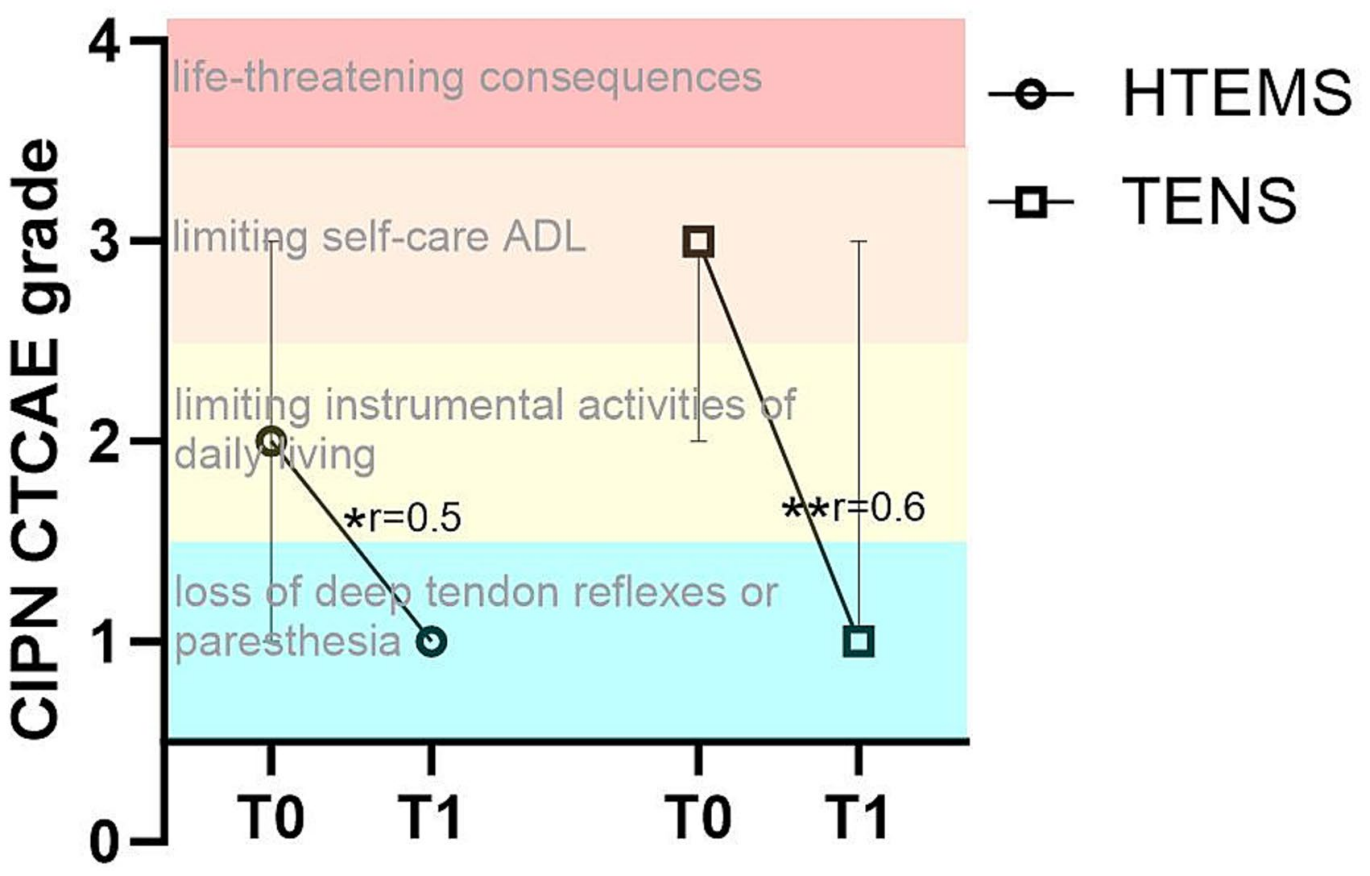
Chemotherapy-induced polyneuropathy (CIPN) grade according to CTCAE v 4 in the transcutaneous electrical nerve stimulation (TENS) and the high-tone external muscle stimulation (HTEMS) groups at baseline (T0) and after the intervention (T1). ADL: Activities of daily living; significant within group differences: ** *p* < 0.01, * *p* < 0.05; r: effect size Pearson’s r; values are presented as median and 95% confidence intervals.

In cancer patients, peripheral neuropathic disorders often occur after the application of certain cytotoxic drugs, especially after taxanes and platinum salts (5). Patients are particularly affected by impairments in sensory functions, e.g., with tingling and numbness of the feet and fingers, which can be still present years after completion of chemotherapy (28, 29). After 8 weeks of home-based electrotherapy, our study showed a significant improvement in sensory and motor functions (Figure 2; Table 2). The CIPN grade according to CTCAE v4 also improved significantly in the TENS group from grade 3 (restricted basic functions; e.g. dressing and personal hygiene) to grade 1 (loss of deep tendon reflexes or paresthesia) and in the HTEMS group from grade 2 (impaired functional tasks; e.g. preparing food or housekeeping) to grade 1 (Figure 3; Supplementary Table S1). These changes are clinically relevant and have a direct impact on the everyday skills of affected patients.

At baseline, patients in our study showed considerably compromised sensory functions according to the EORTC-QLQ-CIPN20 questionnaire (TENS: 45/100, HTEMS: 47/100, CON: 36/100). Eight weeks of electrical therapy led to a significant relief of −12 points (38%) after TENS and − 15 points (45%) after HTEMS therapy, compared to the control group (−3 points, 10%). These improvements were far beyond the minimal clinical important difference of 5.9 points defined by Yeo et al. (22) and did not differ between the two intervention groups. However, only the pre-post changes between HTEMS and the control group differed statistically significantly. A comparison with previous research in electrotherapy as treatment for CIPN is challenging, since the few existing studies are very heterogeneous regarding the population, outcome measures and treatment delivery. One study, where patients received TENS therapy during chemotherapy, reported no beneficial effects (30) while improvements of 10–20% in sensory symptoms were observed by other authors when TENS was performed at least 3 months after stopping chemotherapy (14, 31, 32).

Deterioration of sensory and motor functions after chemotherapy are associated with reduced quality of life and a lower global health status (e.g., according to the EORTC-QLQ-C30), which worsens with symptom intensity (28, 33). A tendency for an increase in the global health status and an improvement for physical functioning after HTEMS were observed in our study. It is nevertheless surprising that the meaningful improvements of sensory impairments after HTEMS in our population were not reflected by clearer increases in quality-of-life domains or the global health status.

Short and easy to perform questionnaires like the CTCAE v4 to assess the CIPN grade are important in clinical routine. This questionnaire includes the limitations of the activities of daily living, is strongly correlated with the EORTC-QLQ-CIPN20 scores and shows a good convergent validity with the EORTC-QLQ-CIPN20 questionnaire (23). After 8 weeks of electrotherapy patients showed a significantly lower CIPN grading in the CTCAE v4 with no differences between intervention groups. However, it should be noted that the improvements after the CTCAE v4 questionnaire have to be viewed with caution since this data was not collected in the control group. Longitudinal studies assessing the course of CIPN show an increase of the incidence of CTCAE grade ≥ 1 and sensory impairments together with successive worsening of symptoms up to 1 year after chemotherapy (34). Spontaneous improvements in sensory impairments or the CTCAE grading without intervention seem therefore unlikely and emphasize the found effects of electrotherapy in our study.

Interestingly, we did not observe changes in any clinical assessment (e.g., cold/warm sensibility, reflexes, tuning fork test, etc.; see Supplementary Tables S2–S4). This is in line with other research reporting only slight changes in quantitative sensory parameters after exercise programs (35). The difference between clinically relevant improvements in CIPN scores vs. the lack of objective improvements by clinical examinations remains to be explained. Insensitivity of clinical evaluations regarding individual neuropathic symptoms but their interplay toward reduced complex neurological functions might be one explanation. Assessments that relate more to daily life activities, like closing buttons or walking on an uneven surface, may be more relevant for patients and may better illustrate impaired sensory and motor functions. Similarly, it is well-known that even relevant differences in specific adverse events of anticancer therapies like nausea or diarrhea do not always cause differences in quality of life in clinical trial patients (36).

### 4.1 Limitations and strengths

When interpreting the results of this study, some limitations have to be considered. One limitation is the decision not to implement a placebo group. The selected intensity of electrotherapy should produce muscle contractions, which would not be achievable using a placebo device. It is therefore possible that patient reported outcomes were biased by the placebo effect. Another limitation is the retrospective recruitment of the control group, which occurred approximately 7 months after the original study protocol. This group was not stratified according to the neurotoxic agent, causing a slight imbalance in the use of platinum compared to the randomized groups, and only completed the EORTC-QLQ-CIPN 20 and C30 questionnaires. The retrospective nature of the control group is a major limitation of our study, but supports the improvements of the sensory scale, especially after HTEMS. Increases in the CTCAE grading after electrotherapy on the other hand, must be interpreted with caution. Deviating from the original study protocol, patients were included with a CIPN grade ≥ 1 according to CTCAE v4. This is in contrast with other research where only patients with CIPN grade ≥ 2 were included. However, despite this difference in the inclusion criteria, patients in our study showed remarkable impairments of sensory and motor functions at baseline measured with the EORTC-QLQ-CIPN20 questionnaire. Finally, ongoing chemotherapy is potential confounder in our trial, since it could have weakened the effect of electrotherapy. Self-evidently, only non-neurotoxic chemotherapies were allowed and the number of patients who received further chemotherapy did not differ between the two groups. As ongoing treatments are common in oncology, prohibiting additional therapies would have not accurately reflected real-world practice. One of the strengths of this clinical trial was - aside from the prospective design and the randomization - the home-based intervention approach. After one extensive training session, patients were able to perform the electrotherapy independently. The treatment was easy and quick to comprehend and travel to a therapy center was not necessary. This was mirrored by a good acceptance and compliance (read from the records of the devices) of 88% (HTEMS) and 80% (TENS). The electrotherapy procedures could be performed safely and there were only a few (16%) unpleasant side effects such as pain caused by too high intensity or discomfort caused by overly adhesive electrodes.

## 5 Conclusion

Electrotherapy, especially the HTEMS intervention, seems to be a successful treatment strategy to mitigate the impairment of sensory functions in CIPN patients. Further investigation is necessary to explore the impact of electrotherapy on everyday tasks and activities. Using a larger sample size and a multicenter approach, the influence of different neurotoxic agents on TENS and HTEMS therapy effects could also be clarified. Although more studies are desirable, HTEMS and TENS can be considered as treatment options for CIPN after completion of neurotoxic chemotherapy since they are easy to administer and have negligible side effects.

## Data Availability

The raw data supporting the conclusions of this article will be made available by the authors, without undue reservation.

## Footnotes

^1^ https://www.sealedenvelope.com/simple-randomiser/v1/lists
